# States of curiosity and interest enhance memory differently in adolescents and in children

**DOI:** 10.1111/desc.13005

**Published:** 2020-07-18

**Authors:** Yana Fandakova, Matthias J. Gruber

**Affiliations:** 1 Center for Lifespan Psychology, Max Planck Institute for Human Development, Berlin, Germany; 2 Cardiff University Brain Research Imaging Centre (CUBRIC), School of Psychology, Cardiff University, United Kingdom

**Keywords:** curiosity, motivation, interest, surprise, learning, memory, children, adolescents

## Abstract

Curiosity - broadly defined as the desire to acquire new information - enhances learning and memory in adults. In addition, interest in the information (i.e., when the information is processed) can also facilitate later memory. To date, it is not known how states of pre-information curiosity and post-information interest enhance memory in childhood and adolescence. We used a trivia paradigm in which children and adolescents (N = 60, 10–14 years) encoded trivia questions and answers associated with high or low curiosity. States of high pre-answer curiosity enhanced later memory for trivia answers in both children and adolescents. However, higher positive post-answer interest enhanced memory for trivia answers beyond the effects of curiosity more strongly in adolescents than in children. These results suggest that curiosity and interest have positive effects on learning and memory in childhood and adolescence, but might need to be harnessed in differential ways across child development to optimize learning.

## Introduction

A fledgling research field on curiosity has suggested that intrinsic states of curiosity – the desire to acquire new information – enhances learning and memory (for reviews, see Gruber et al., 2019; Gruber & Ranganath, 2019). In line with these findings, neuroimaging studies in adults have demonstrated that ‘pre-information’ curiosity states elicit increased neural activity in memory- and reward-related brain regions, including the hippocampus and the striatum, respectively (Charpentier et al., 2018; Gruber et al., 2014; Jepma et al., 2012; Kang et al., 2009; Lau et al., 2020; Ligneul et al., 2018; Oosterwijk et al., 2019). These activity increases associated with pre-information curiosity predict the beneficial effects of curiosity on later memory performance (Gruber et al., 2014).

In addition to pre-information curiosity, the situational interest associated with the actual information (i.e., post-information interest) may also play an important role for learning (Fastrich et al., 2018; Ligneul et al., 2018; Marvin & Shohamy, 2016; McGillivray et al., 2015). Situational interest is conceptualized as a motivational variable that triggers feelings of enjoyment and a situational rise in attention sparked by specific features of the information (Grossnickle, 2016; Hidi & Renninger, 2006; Renninger & Hidi, 2015). Consistent with findings that situational interest positively influences learning in educational settings (Renninger & Hidi, 2015; Schiefele et al., 1992), recent experimental studies with adults have demonstrated that higher interest for presented information is associated with enhanced learning and memory (Fastrich et al., 2018; Marvin & Shohamy, 2016; McGillivray et al., 2015).

Even though the available evidence suggests that both pre-information curiosity and post-information interest benefit adults’ learning and memory, relatively less is known about how the two constructs are related and how they jointly drive learning. While the two constructs have rarely been investigated together in the context of the same study (Grossnickle, 2016; but see Fastrich et al., 2018; McGillvray et al., 2015), it has recently been proposed that so-called information prediction errors (IPEs) - the discrepancy between post-information interest and pre-information curiosity - may be an important driver of memory (Marvin & Shohamy, 2016). The authors showed that IPEs modulated memory in adults such that participants were more likely to remember information when it was associated with more positive IPEs (i.e., information that was judged more satisfying or interesting than the initial level of pre-information curiosity). These additional effects of positive IPEs on memory have subsequently been replicated in recent studies with adults (Fastrich et al., 2018; Ligneul et al., 2018).

The concept of IPEs represents a critical aspect of the information gap theory (Loewenstein, 1994) which postulates that predicting whether the expected information will resolve uncertainty is critical for curiosity (Marvin & Shohamy, 2016; Oudeyer et al., 2007). IPEs are also in line with the theoretical ideas and extensive evidence on reward prediction errors - the difference between a received reward value and the originally expected reward value (Rescorla & Wagner, 1972; Schultz et al., 1997; Schultz, 2013). Reward prediction errors have been shown to be a key driver of learning in various contexts (Rescorla & Wagner, 1972; Schultz, 2013). Consistent with the recent findings of positive IPEs on memory, positive reward prediction errors also have beneficial effects on episodic memory in adults (e.g., Jang et al., 2019; Pine et al., 2018; for a review, see Ergo et al., 2020). Taken together, both pre-information curiosity and post-information interest may contribute to learning success, and IPEs might be a fruitful approach to investigate how higher than initially expected situational interest might affect memory (Fastrich et al., 2018; Ligneul et al., 2018; Marvin & Shohamy, 2016).

But how do curiosity, interest, and IPEs affect memory in children? Especially in school settings, curiosity has been praised for its positive effects on learning and teachers have been encouraged to stimulate curiosity in the classroom (Engel, 2011; Hidi & Renninger, 2006; Montessori, 1948; Oudeyer et al., 2016). Experimental research shows that already infants and young children explore and attend to the world around them in a way that is consistent with a ‘curiosity drive’ to close knowledge gaps and to reduce uncertainty (Kidd & Hayden, 2015; Schulz, 2012). For example, infants explore information guided by their own curiosity (Begus et al., 2014, 2016) and prefer material of intermediate complexity (Kidd et al., 2012). Materials of intermediate complexity are expected to show highest curiosity levels according to the information gap theory (Loewenstein, 1994; Oudeyer et al., 2007) and adults express highest states curiosity for information associated with intermediate levels of knowledge confidence (Baranes et al., 2015; Kang et al., 2009). Preschoolers prefer to play with toys for which they do not completely understand the underlying mechanism (Schulz & Bonawitz, 2007), suggesting that young children explore their environment to enhance information gain. However, while these findings are well aligned with the idea that curiosity is important for learning in infancy and young childhood, most developmental studies documenting that children seek out information have not assessed curiosity directly. Thus, there exists a gap in the literature between the proposed importance of curiosity for learning in childhood (Renninger & Hidi, 2019; Engel, 2011; Gottlieb et al., 2013; Jirout et al., 2018) and empirical evidence directly assessing curiosity states and their benefit for learning and memory in children. While previous research has indicated that curiosity as an individual trait facilitates learning in educational contexts (Kashdan & Yuen, 2007; Shah et al., 2018; von Stumm et al., 2011), it is currently unknown how states of curiosity affect learning and memory in children and adolescents. As states of curiosity are potentially more malleable than trait curiosity, a better understanding of the effects of curiosity in development can help facilitate educational practices related to fostering children’s and adolescents’ learning.

In contrast, interest and its development has been an active focus of research in children, especially in educational settings (Hidi & Renninger, 2006; Renninger & Hidi, 2015). This research field has shown that the affective and cognitive processes associated with interest change across development. Students’ self-reported levels of interest in different academic domains has been shown to decrease with age (Fredricks & Eccles, 2002; Frenzel et al., 2012; Hidi & Harackiewicz, 2000). Interest for a given domain also changes within a person over time such that emotional components dominate early stages of interest development, whereas cognitive components dominate later stages of interest development (Hidi & Renninger, 2006). Similar trends have been also observed across child development. For example, one longitudinal study demonstrated that children shifted from more affective to more cognitive aspects of interest in mathematics between 10-11 years and 14-15 years (Frenzel et al., 2012). However, research on the development of interest in childhood and adolescence has focused primarily on individual interest, or the enduring disposition towards information in a particular domain (Krapp, 2002; Silvia, 2006). In this context, individual interest in an academic domain has been found to be an important predictor of academic achievement (Hidi & Renninger, 2006; Schiefele et al., 1992). By contrast, the effects of situational interest on learning across childhood and adolescence have received relatively less attention in research and have mostly focused on features that make specific topics more interesting (cf. Hidi & Harackiewicz, 2000). Understanding how situational interest affects memory performance across development would help clarify how the closing of an existing information gap modulates learning.

Compared to younger children and adults, adolescents are more sensitive to extrinsic rewards due to enhanced modulation of reward-related brain regions (Blakemore & Robbins, 2012; Galvan et al., 2006; Somerville et al., 2010; Somerville & Casey, 2010). In one study, adolescents showed better memory for pictures associated with positive reward prediction errors (i.e., receipt of higher monetary reward than initially expected) suggesting that increased reward sensitivity benefits learning during adolescence (Davidow et al., 2016; see also, Hallquist et al., 2018; van den Bos et al., 2012). These findings corroborate similar findings in adults demonstrating that greater and more positive prediction errors enhance episodic memory (Ergo et al., 2020). As the effects of prediction error associated with intrinsically valuable information on memory have only recently been demonstrated in adults (Fastrich et al., 2018; Ligneul et al., 2018; Marvin & Shohamy, 2016), it is an open question to what extent they are also present in childhood and adolescence. The findings of age differences in the effects of extrinsic rewards on learning between childhood and adolescence (van den Bos et al., 2012; van Duijvenvoorde et al., 2016) suggest that adolescents may show a more adult-like pattern and benefit more from encountering information that is more interesting than initially expected as measured by IPEs, or the discrepancy between post-answer interest and pre-answer curiosity. Such a pattern of age differences would also be consistent with ideas that children bring along greater energy to explore and learn various new skills whereas adolescence is associated with few, selected fields of interest (cf. Frenzel et al., 2012).

In contrast to these studies on age differences in extrinsic rewards, the highlighted findings from infants and younger children that the effects of uncertainty reduction and exploration of material with manageable complexity are present already early on leave open the question whether encountering higher than originally expected interesting information entails uniform memory benefits across child development or, as suggested by the highlighted findings on reward, there may be differences between children and adolescents. The current study aims to (i) close this knowledge gap on the effects of situational curiosity and interest on memory between childhood and adolescence, and (ii) examine the memory effects of higher than initially expected interesting information (i.e., positive IPEs).

### The Present Study

**Figure 1:**
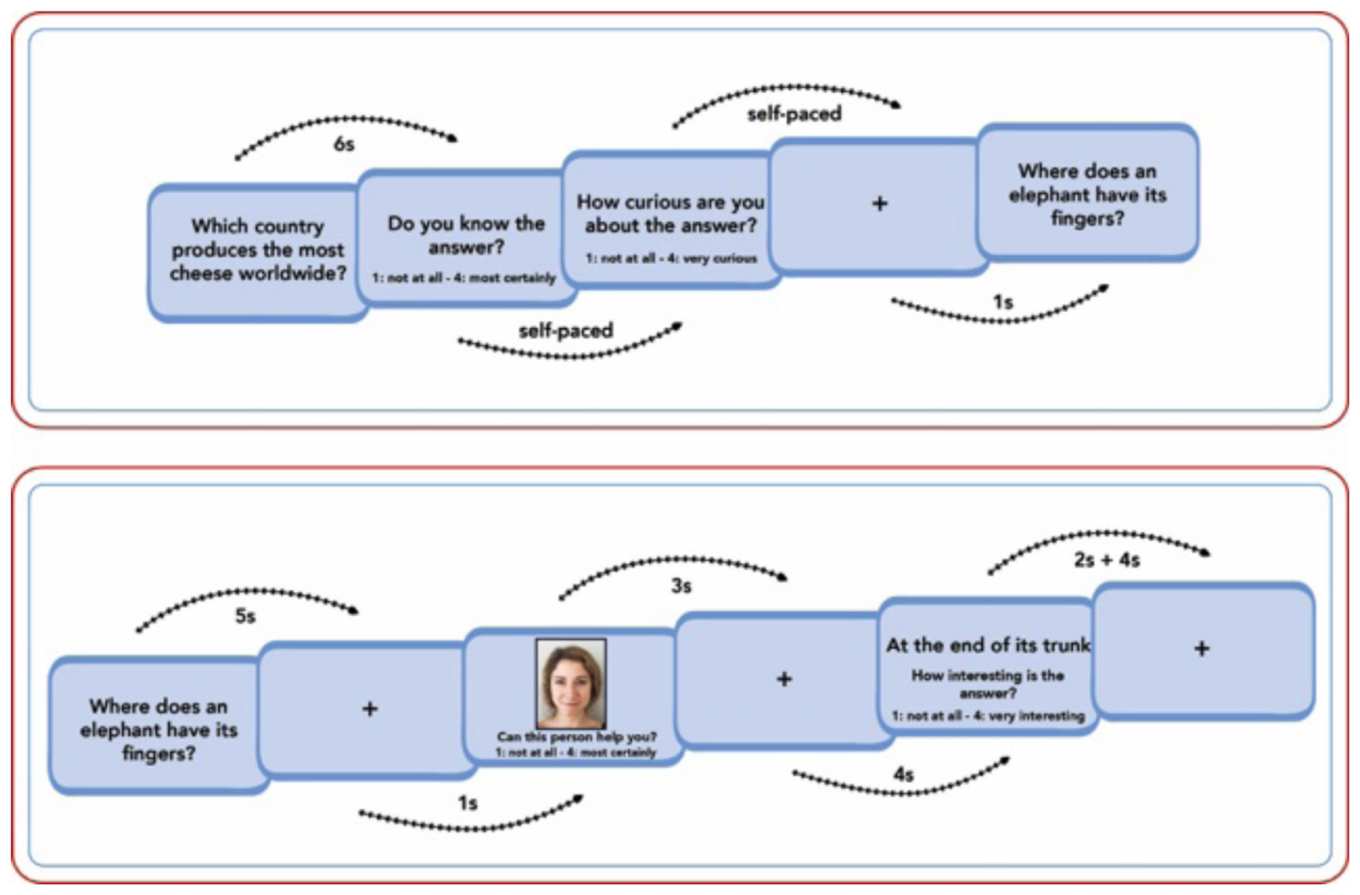
Experimental paradigm. Upper panel represents the screening phase in which we selected participant-specific trivia questions for which the answers were unknown and which varied in subjective curiosity. Lower panel represents the subsequent study phase of the experiment. Here, a trivia question was presented and participants anticipated the correct answer over a delay of 13 seconds. During the anticipation phase, we presented an incidental image of an adult face to investigate potential memory enhancements for incidental information encountered during high-curiosity states. After the correct answer was shown, participants rated its interestingness on a 4-point scale.

We investigated how curiosity and interest separately and jointly via IPEs contribute to memory in children and adolescents between 10 and14 years. To induce curiosity, we used a trivia paradigm in which participants anticipated answers to trivia questions that were associated with varying degrees of curiosity (Figure 1). Studies with adults have consistently shown that answers to trivia questions associated with high curiosity are better remembered than answers to low-curiosity questions (Fastrich et al., 2018; Galli et al., 2018; Gruber et al., 2014; Kang et al., 2009; Marvin & Shohamy, 2016; McGillivray et al., 2015; Mullaney et al., 2014; Stare et al., 2018; Wade & Kidd, 2019). First, participants rated their curiosity about a series of trivia questions. Subsequently, participants anticipated and encoded the correct answer. During the anticipation phase, participants encoded an incidental face image to investigate potential memory enhancements for incidental information encountered during high-curiosity states (as has been shown for adults: Galli et al., 2018; Gruber et al., 2014; Stare et al., 2018). Following the presentation of the answer participants rated their subjective interest in the answer. Memory for the faces presented during anticipation was tested after a 20 min delay, followed by a cued-recall test of the answers to the trivia questions.

The design, predictions, and planned analyses were preregistered on Open Science Framework. We had the following key predictions about the effects of curiosity and interest on memory: (1) Children and adolescents would demonstrate a curiosity-related memory enhancement for trivia answers and potentially incidental face images associated with high-compared to low-curiosity trivia questions. (2) Higher post-answer interest ratings would be associated with enhanced memory in children and adolescents. (3) We predicted that positive IPEs (i.e., a greater discrepancy between initial curiosity and post-answer interest) would result in enhanced memory for an answer, and that IPE effects on memory would be larger in adolescents than in children.

## Methods

### Participants

As outlined in the OSF preregistration, we aimed to acquire a total of *N* = 30 complete data sets from children (10.06–12.99 years, *M*_*age*_ = 11.37 years, *SD*_*age*_ = 0.81 years; 15 females) and *N* = 30 adolescents (13.11–14.99 years, *M*_*age*_ = 14.05 years, *SD*_*age*_ = 0.64 years; 15 females). The planned sample size for both groups was based on the adult sample size in Gruber et al. (2014), which used the same paradigm. Data sets were considered complete when data from all four experimental phases were available. In total, 69 children were recruited from the database of the Max Planck Institute for Human Development in Berlin, Germany. Data of six participants were excluded due to technical problems that resulted in participants seeing certain stimuli more than once or not at all. Two additional participants did not complete all four phases of the experiment, and one participant was excluded due to non-compliant behavior during the memory test. Children were native German speakers (i.e., German is the main language spoken at home), had no history of neurological or psychiatric disorders, were not born prematurely (before 37th week of pregnancy) and had normal or corrected-to-normal vision. Families received 25 Euros for their participation in the study. The study was approved by the local ethics committee of the Max Planck Institute for Human Development.

### Materials

#### Trivia questions and answers

We generated a pool of 445 trivia questions along with their corresponding answers from online trivia websites (see https://osf.io/5tp8j/ for a full list of the questions). The questions belonged to trivia categories expected to elicit different levels of curiosity in children: computer games and media, geography and history, science and medicine, religion and politics, general knowledge, sports, languages and books, art and music. The pool contained trivia questions for which the answers were likely to be unknown to the majority of participants.

#### Faces

Each picture showed the face of an adult with a neutral face expression, in front of a naturalistic background. A total of 90 faces were divided into three subgroups of 30 stimuli each, which were counterbalanced across participants for the following three experimental components: the high- and low-curiosity conditions as well as new faces for the surprise recognition test.

#### Post-experimental questionnaires and eye-tracking

To explore the extent to which potential curiosity-related and IPE-related memory enhancements were associated with individual variability in personality characteristics related to curiosity, participants completed a set of questionnaires at the end of the experiment (see OSF preregistration for details, https://osf.io/qyf9m/). These measures have not been analyzed for this manuscript.

### Task Procedures

Participants underwent a four-stage paradigm with (1) a screening phase, (2) a study phase, (3) a ∼20-minute delayed surprise recognition test phase for incidental face images, and (4) a subsequent surprise recall test for trivia answers presented during the study phase (Figure 1). The Cogent 2000 toolbox (http://www.vislab.ucl.ac.uk/cogent.php) was used for all experimental phases. In all four phases of the experiment, stimuli were presented on a gray background in the center of the computer screen.

#### (1) Screening phase

Because the level of curiosity elicited by different trivia questions is likely to vary between participants, we used participants’ ratings to sort trivia questions into participant-specific high- and low-curiosity categories (30 questions each). Trivia questions were randomly selected from the aforementioned pool and were consecutively presented on the screen. After a trivia question was presented for 6 s, participants were instructed to give two self-paced ratings on four-point scales. First, they rated how confident they were that they knew the answer to a trivia question (“Do you know the answer?” [Weisst du die Antwort?]; extreme points: 1 = “no idea” [keine Ahnung] and 4 = “pretty sure” [ziemlich sicher]). Second, participants rated their level of curiosity about the answer to a trivia question (“How curious are you about the answer?” [Wie neugierig bist du auf die Antwort?]; extreme points: 1 = “not curious at all” [gar nicht neugierig] and 4 = “very curious” [sehr neugierig]). After a response was given for the second rating, an inter-trial cross hair was presented for 1 s. If participants did not indicate that they knew the answer to a trivia question by rating their confidence with a 4, trivia questions with responses 1 or 2 to the curiosity rating were allocated to the low-curiosity condition and responses 3 and 4 were allocated to the high curiosity condition. If participants rated their confidence that they knew the answer with a rating of 4, the question was discarded and not used in later phases of the experiment. For each participant, the screening phase lasted until 30 trivia questions had been allocated to each curiosity condition.

#### (2) Study phase

In the subsequent study phase, the selected 60 trivia questions were presented along with the associated answers. A trial started with the presentation of a trivia question for 5 s, followed by an anticipation period of 13 s. During the anticipation period (i.e. from the onset of the trivia question to the onset of the trivia answer), a cross hair was presented after the trivia question. The cross hair was replaced by an emotionally neutral adult face (incidental item) from 6 to 9 s after the onset of the trivia question. During the presentation of the face, participants were instructed to judge on a four-point scale as to whether the person depicted on the image could help them figure out the answer (“Can this person help you?” [Kann dir diese Person helfen?]; extreme points: 1 = “not at all” [gar nicht] and 4 = “most certainly” [auf jeden Fall]). This encoding judgment ensured that faces were likely to be encoded with a similar level of attention across both curiosity conditions.

After the presentation of the trivia answer for 2 s, a post-answer interest rating was presented for 4 s (“How interesting is the answer?” [Wie interessant ist die Antwort?]; extreme points: 1 = “not interesting at all” [gar nicht interessant] and 4 = “very interesting” [sehr interessant]). Subsequently, a cross hair was again presented during the inter-trial interval, which was temporally jittered for 4–4.5 s. 10% of the trials in each condition (3 out of 30 trials) were catch trials in order to ensure participants’ attention throughout the phase. In these trials, the letter string ‘xxxxx’ was presented instead of the trivia answer. We divided the study phase into four blocks (15 trials each). After the study phase, children played board games with the experimenter that were not related to the task in any way.

#### (3) Recognition memory test for incidental items

Approximately 20 min after the end of the study phase, participants took part in a surprise recognition memory test for the incidental face images. During the break between the phases, children played games with the experimenter that were not related to the task (e.g., UNO, Connect4). All 60 faces from the study phase and 30 new faces were randomly presented in consecutive order. Each face was presented for 3 s. Participants had to decide whether they were confident that the face image had been presented during the earlier study phase or it was novel (i.e., “confident new” [sicher neu], “unconfident new” [nicht so sicher neu], “unconfident old” [nicht so sicher alt], and “confident old” [sicher alt]). Participants were encouraged to try to give a response as accurately and quickly as possible. The inter-trial interval displaying a cross hair was temporally jittered with a 5–5.5 s duration.

#### (4) Recall test for trivia answers

Immediately following the recognition memory test for incidental items, participants were presented with all trivia questions from the study phase in random order. A question was presented on the screen and participants were asked to verbally recall the answer or to say “I don’t know” [Weiss ich nicht] if they did not remember the answer to a trivia question. We discouraged the guessing of answers. The experimenter recorded the participants’ answers on an Excel sheet and then proceeded to present the next question on the screen.

In all phases, responses on the four–point scale were given on a computer keyboard using the left and right middle and index fingers. Prior to each experimental phase, participants were instructed for the upcoming phase and practiced on items that were not used in the main task to ensure that they used the rating categories correctly. After all experimental phases were completed, participants filled out different post-experimental questionnaires (see details in OSF preregistration). The whole visit to the laboratory lasted approximately 2.5 hours.

#### Eye-tracking

Eye gaze and pupil dilations were continuously recorded on a subset of children (N = 46) throughout the study phase. In addition, we recorded spontaneous eye-blink rates in short sessions at three time points during the experiment: prior to the screening phase (5 min), between the screening and study phase (3 min), and following the study phase (3 min). These data were not analyzed for the present manuscript.

### Behavioral Analyses

We used ANOVAs to examine age differences between children (10–12 years, *N* = 30) and adolescents (13–14 years, *N* = 30) in the effects of pre-answer curiosity (high-curiosity vs. low-curiosity condition) and post-answer interest (high vs. low post-answer interest) on memory recall as a dependent variable. The high-curiosity condition included “3” and “4” curiosity ratings, and the low-curiosity condition included “1” and “2” curiosity ratings. The high post-answer interest condition included interest ratings “3” and “4”, and the low post-answer interest condition included ratings “1” and “2”. Note that per experimental design (cf. *Screening phase*) every participant encountered 30 questions in each of the high-curiosity and low-curiosity conditions, whereas the number of questions in the high vs. low post-answer interest categories varied depending on participants’ responses. There were no differences between the proportion of high vs. low post-answer interest categories between the age groups, t(53.9) = 0.43, p = .67 (high post-answer interest in children M = 56.65%, SD = 15.73%, in adolescents M = 58.26%, SD = 13.26%).

To examine how states of curiosity modulated memory for incidental information (i.e., faces), we computed face recognition accuracy as hits (i.e., a confident or unconfident “old” response to a studied face) minus false alarms (i.e., a confident or unconfident “old” response to a novel face; (Snodgrass & Corwin, 1988). We performed ANOVAs to examine age differences (children vs. adolescents) in face recognition for faces presented following high-curiosity (i.e., “3” and “4” curiosity ratings) vs. low-curiosity (“1” and “2” curiosity ratings) conditions. Follow-up exploratory analyses examined curiosity effects on face memory separately for “confident” and “unconfident” responses during face recognition.

All computations were performed in R version 3.6.3 (R Core Team, 2020). ANOVAs were performed using the ezANOVA package (https://cran.r-project.org/web/packages/ez/ez.pdf). For all ANOVAs we divided the sample into child and adolescent groups, consistent with the initial study design. Given the continuous age range in the present study, we followed up on significant main or interactive effects of age group with correlation analyses (i.e., *Pearson’s r*, one-sided) treating age as a continuous variable to confirm that results were not driven solely by the group split. False discovery rate (FDR) corrections were applied for multiple comparisons in all reported analyses (labeled *p*_*adj*_ ).

Finally, we examined the effects of the discrepancy between the actual value of the presented information relative to the participants’ initial curiosity expectation on memory for trivia answers. To this end, an IPE score was computed for each trial as the difference between the initial curiosity rating and the post-answer interest rating (Marvin & Shohamy, 2016). To examine the interactive effects of curiosity and IPE, we performed linear mixed-level analyses on trial-level data (cf. Marvin & Shohamy, 2016; McGillivray et al., 2015). Mixed-effect models allow within-person examination of curiosity and IPE effects with more fine-grained distinctions between levels of curiosity and IPE, while at the same time accounting for variability across participants. To ensure consistency of results, we also tested a model in which rather than focusing on curiosity and IPE, we examined curiosity and interest ratings on the trial level.

Mixed-effects models were implemented using the brms package (Bürkner, 2017), which allows for fitting a wide range of models. Mixed effects logistic regressions with trivia answer accuracy as the dependent variable were fit to single-trial data. In a first model, curiosity ratings, IPE scores, and participants’ age were used as independent variables. This approach has been used previously to investigate the influence of IPEs on memory in adults (Marvin & Shohamy, 2016; Fastrich et al., 2018). In a second model, curiosity ratings, post-answer interest ratings, and participants’ age were used as independent variables. Trial-based data (i.e., curiosity ratings, IPE scores, post-answer interest ratings) were z-transformed within individuals. Age was z-transformed across the entire study sample (Mage = 12.7 years). For all models, we included a by-subject random intercept and a random slope for each of the main effects considered in the model (cf. Barr et al., 2013). The fitted models included four chains of 5000 iterations each, excluding a warm-up period of 5000 iterations. Models were fit with default priors using the bernoulli family distribution. All chains indicated convergence, according to the Gelman-Rubin rhat statistic (rhat < 1.01). We report the estimate and its associated error for all models, and summarize the posterior distributions with equally tailed 95% credible intervals (CrI). The hypothesis function in brms was used to compute a posterior probability for each effect (Bürkner, 2017). For two-sided hypotheses, the evidence ratio and corresponding posterior probability is based on a Bayes factor between the hypothesis and its alternative computed via the Savage-Dickey density ratio method. The size of the credible interval was specified at 95% for all analyses.

## Results

### Does pre-answer curiosity modulate memory for trivia answers in childhood?

**Figure 2:**
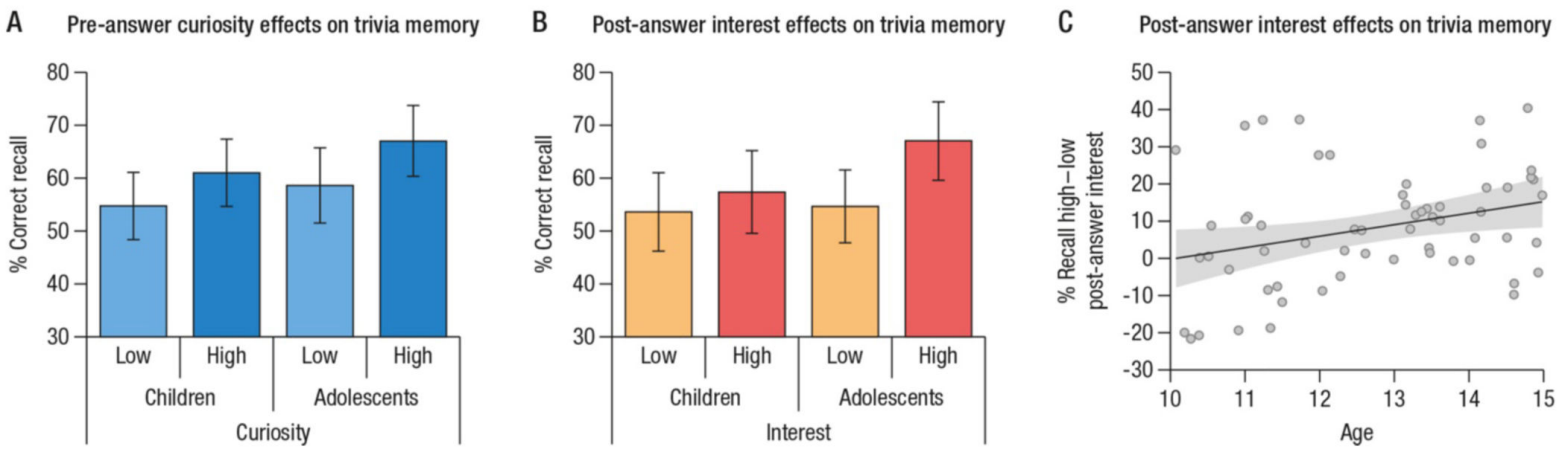
**A**. Pre-answer curiosity-related effects on memory for trivia answers in children and adolescents **B**. Post-answer interest effect on memory for trivia answers in children and adolescents **C**. Correlation between participants’ age and interest-driven memory advantage for trivia answers. Error bars (A, B) and shaded area (C) show 95% confidence intervals. High curiosity or interest is defined as ratings “3” or “4” on the corresponding scale, low curiosity or interest is defined as ratings “1” or “2” on the corresponding scale.

An ANOVA on the proportion of correctly recalled trivia answers with the within-subjects factor pre-answer curiosity (high vs. low) and the between-subjects factor age group (children vs. adolescents) revealed a significant main effect of curiosity (*F*_(1, 58)_ = 26.02, *p* < .001, *η*_*p*_^*2*^ = .31, M_difference_ = 7.4%). There was neither a significant main effect of age group (*F*_(1, 58)_ = 1.31, *p* = .26, *η*_*p*_^*2*^ = .02) nor a significant curiosity-by-age group interaction (*F*_(1, 58)_ = 0.53, *p* = .47, *η*_*p*_^*2*^ = .01) (Figure 2A). These results suggest that curiosity did indeed enhance memory for trivia answers, and the enhancement effect was similar in children and adolescents.

### Does post-answer interest modulate memory for trivia answers in childhood?

An ANOVA on the proportion of correctly recalled answers with the within-subjects factor post-answer interest (high vs. low) and the between-subjects factor age group (children vs. adolescents) revealed a main effect of interest (*F*_(1,58)_ = 17.60, *p* < .001, *η*_*p*_^*2*^ = .23) along with a significant age group-by-post-answer interest interaction (*F*_(1,58)_ = 4.92, *p* = .03, *η*_*p*_^*2*^ =.08). The main effect of age group was not significant (*F*_(1,58)_ = 1.29, *p* = .26, *η*_*p*_^*2*^ = .02). Paired-sample post-hoc tests within each age group showed that recall did not differ significantly between high-and low post-answer interest in younger children (*t*_(29)_ = 1.19, *p*_*adj*_ = .24, *M*_*difference*_ = 4%). In contrast, older children were significantly more likely to remember answers that received high as compared to low post-answer interest ratings (*t*_(29)_ = 5.73, *p*_*adj*_ < .001; *M*_*difference*_ = 12%) (Figure 2B). In line with the group analyses, the post-answer interest-driven memory enhancement (proportion correct recall for high versus low post-answer interest) was positively correlated with participants’ age (*Pearson’s r* = .31, *p*_*one-tailed*_ = .01; Figure 2C).

### How do information prediction errors modulate memory for trivia answers in childhood?

**Figure 3:**
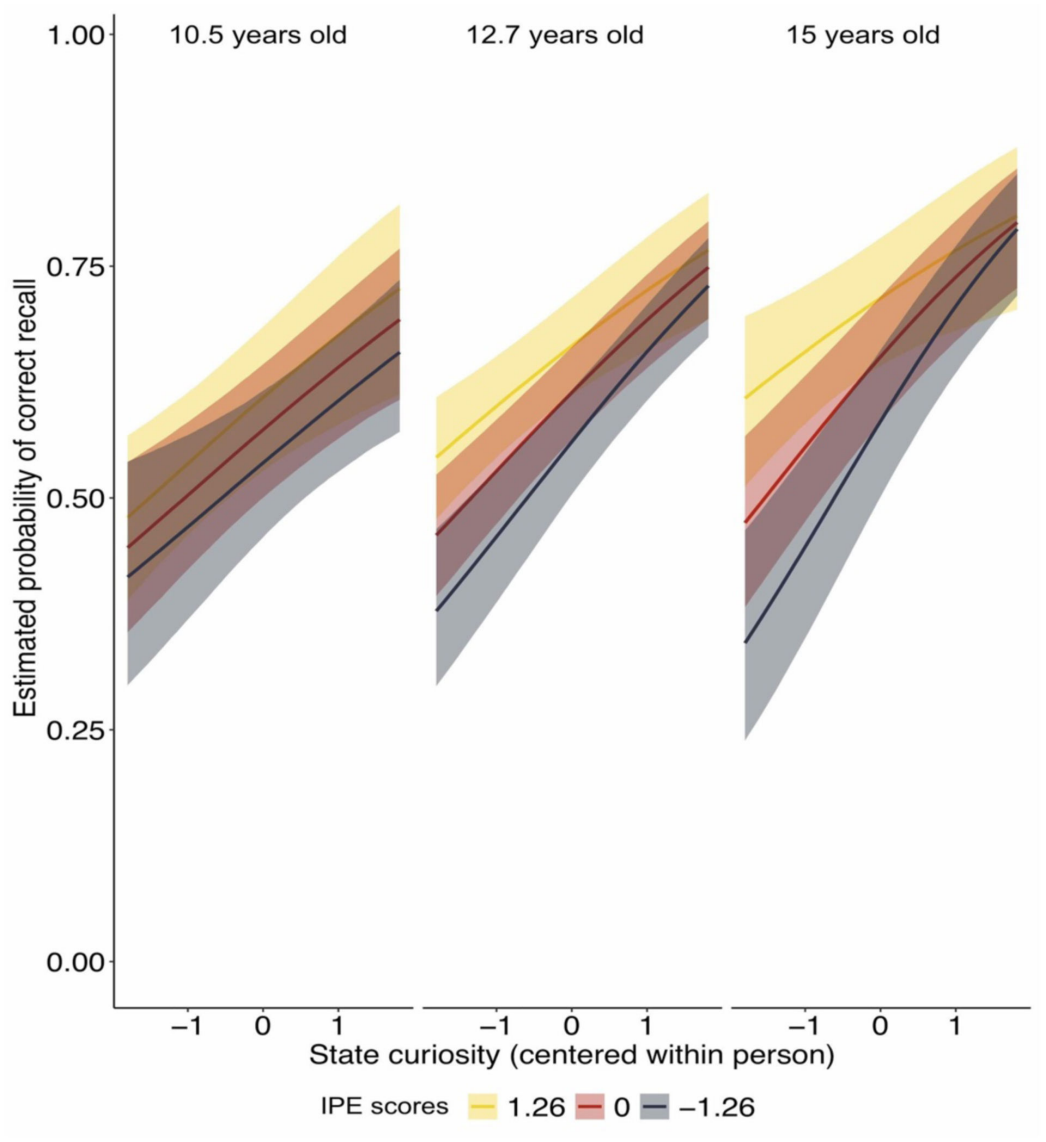
For low-curiosity answers, positive IPEs enhance memory with increasing age. Estimated probability of correct recall is displayed for different levels of curiosity and IPE split by three different example ages that represent the lower end of the sample (10.5 years), the mean age of the sample (12.7 years) and the maximal age in the sample (15 years). Levels of state curiosity and IPE score were centered within person and represent deviations from the person-specific means. Shaded areas indicate 95% credible intervals as estimated in the brms package (Bürkner, 2017). Age was used as a continuous variable in the model, and was split in the figure for display purposes only. IPE = information prediction error.

One possible mechanism by which post-answer interest may modulate the effects of curiosity on episodic memory is via the extent to which it reflects positive or negative discrepancy between the actual value of the presented information relative to the participants’ initial curiosity expectations (i.e., IPEs). To test this, we performed trial-level analyses predicting recall accuracy by (centered within participants) curiosity ratings, IPE scores, and mean-centered age. The results revealed that both curiosity (*95%CrI* = [0.25,0.44], *Post*.*Prob*. = 1) and IPE (*95%CrI* = [0.09,0.25], *Post*.*Prob*. = 1) enhanced the likelihood for correctly recalling a trivia answer in children and adolescents (see Table 1 for full model results). Critically, we also observed evidence for a curiosity-by-IPE-by-age interaction (95%*CrI* = [-0.08, 0.00], *Post*.*Prob*. = 0.97). To unpack this interaction, we examined the effects of IPE, age, and their interaction separately for questions associated with high and low states of pre-answer curiosity. As outlined above, we expected that IPEs - the discrepancy between initial curiosity and post-answer interest - will especially enhance memory for low-curiosity questions. Accordingly, for answers to low-curiosity questions, we observed an effect of IPE on answer memory accuracy (*95%CrI* = [0.07,0.28], *Post*.*Prob*. = 1), along with an IPE-by-age interaction (*95%CrI* = [0.00,0.14], *Post*.*Prob*. = .98) such that older age was associated with a more pronounced effect of IPE on recall of low-curiosity answers (Figure 3; see Table 1 for full model results). In contrast, for answers to high-curiosity questions (Table 1), we observed an effect of age (*95%CrI* = [0.01,0.31], *Post*.*Prob*. = .98) with adolescents showing enhanced memory for answers to high-curiosity questions. However, we found no strong evidence for an effect of IPE or an IPE-by-age interaction (see Table 1 for full model results).

Given that IPE was computed as the discrepancy between curiosity and interest, in analogy to how reward prediction errors have been computed (Marvin & Shohamy, 2016), we sought to further confirm the effects of curiosity and interest on learning by testing a model in which we predicted trivia answers recall with curiosity ratings and post-answer interest ratings as separate factors along with age (cf. Fastrich et al., 2018; McGillivray et al., 2015). The results were consistent with the results based on IPE (see Table 2 for full model results). We observed an effect of interest (*95%CrI* = [0.09,0.25], *Post*.*Prob*. = .1), curiosity (*95%CrI* = [0.09,0.25], *Post*.*Prob*. = .1) as well as age (*95%CrI* = [0.01,0.29], *Post*.*Prob*. = .98) such that higher curiosity, higher post-answer interest and older age were associated with higher likelihood to correctly recall the trivia answer. Moreover, in line with the IPE analyses above, we found evidence for a curiosity-by-interest interaction (*95%CrI* = [-0.17,-0.02], *Post*.*Prob*. = .99), suggesting that effects of post-answer interest were especially stronger when initial curiosity was low (see Fig XX below). Although the interactions with age showed the same patterns as the previous analysis using IPEs instead of interest, we did not find reliable evidence for a curiosity-by-IPE-by-age interaction (*95%CrI* = [-0.07, 0.02], *Post. Prob*. = 0.81).

Taken together, while states of curiosity were associated with enhanced memory in children and adolescents, when curiosity was low, adolescents’ recall benefited to a greater extent than children from encountering a more interesting than initially expected answer (i.e. positive IPE).

### Do children and adolescents show enhanced memory for incidental information presented during high- compared to low-curiosity states?

An ANOVA on recognition memory accuracy [hits – false alarms] for the incidental face images with the factors pre-answer curiosity (high vs. low) and age group (children vs. adolescents) revealed no significant effects of age group, (*F*_(1,58)_ = 2.73, *p* = .10, *η*_*p*_^*2*^ = .05), curiosity, (*F*_(1,58)_ = 0.43, *p* = .52, *η*_*p*_^*2*^ = .01), nor a curiosity-by-age group interaction(*F*_(1,58)_ = 0.58, *p* = .45, *η*_*p*_^*2*^ = .01, Table 3). In a follow-up exploratory analysis, we examined the curiosity effect on incidental information separately for faces recognized with high vs. low confidence. For high-confidence face recognition, we found a main effect of age group (*F*_(1,56)_ = 4.203, *p* = .05, *η*_*p*_^*2*^ = .07), but again no effects of curiosity or a curiosity-by-age group interaction (*all ps >* .*22*). None of the effects reached significance for low-confidence recognition (*all ps* > .72).

**Figure 4:**
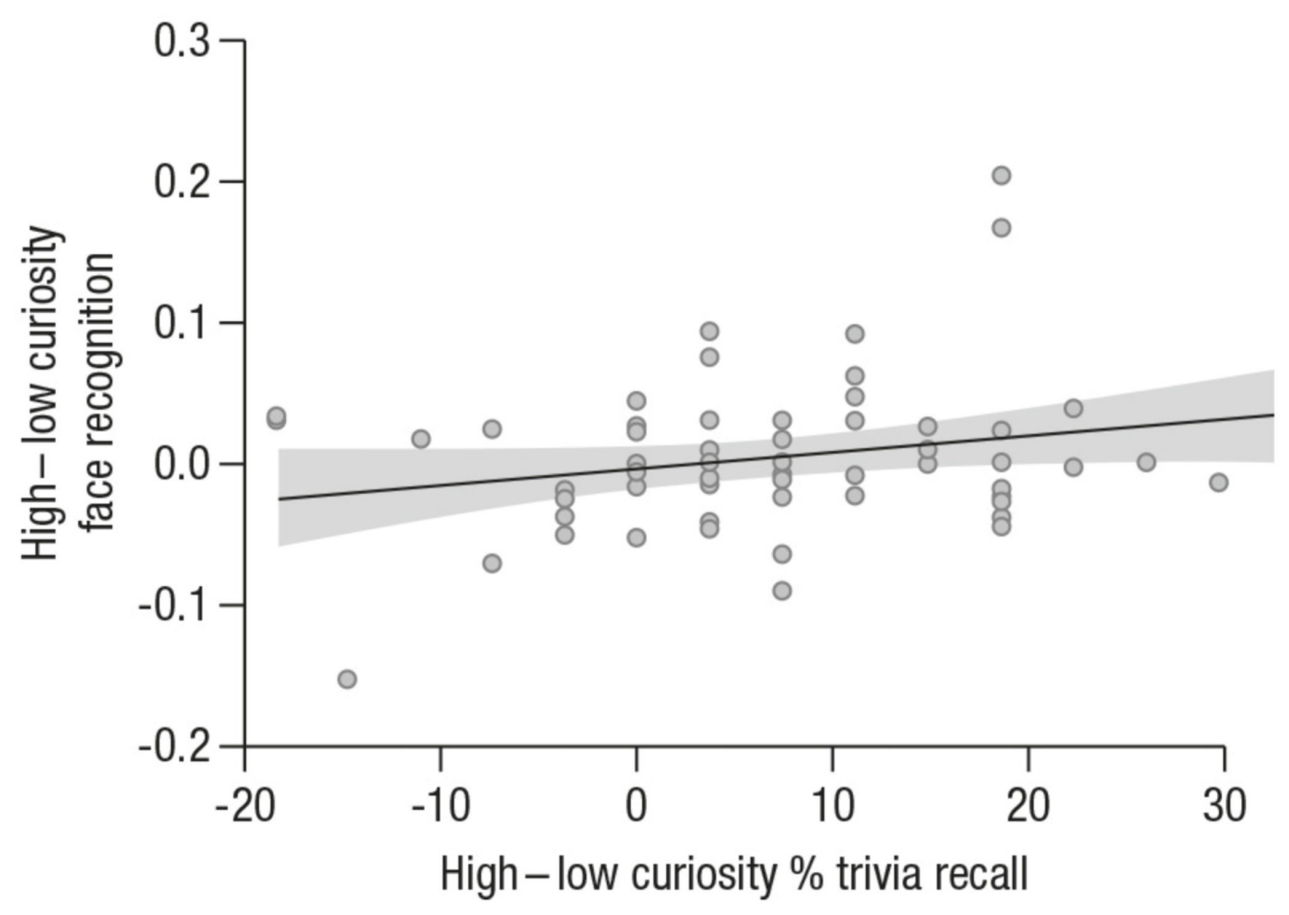
Exploratory correlation between curiosity-related enhancement of face recognition (difference in hits – false alarms for high- vs. low-curiosity) and curiosity-related enhancement of trivia answer recall (difference in % recall for high-vs. low-curiosity).

Based on the hypothesis that enhanced memory for incidental information would reflect a potential spill-over effect from the pre-answer curiosity about the trivia questions, it is possible that the curiosity-based enhancement of face memory was stronger in participants who showed a more pronounced curiosity-based advantage in trivia answer recall. To test this in exploratory analyses, we correlated the curiosity-based enhancement of trivia answer recall (proportion recall for high – low curiosity) with the curiosity-based enhancement of face recognition (memory accuracy for high – low curiosity). The results showed a significant positive correlation (*Pearson’s r* = .23, *p*_*one-tailed*_ = .036; Figure 4) such that children who showed a greater benefit of curiosity for trivia answer recall were also most likely to show an enhancement in face memory between the high- and low-curiosity conditions. Controlling for age did not change these exploratory results, *Pearson’s r* = .23, *p*_*one-tailed*_ = .04.

## Discussion

The present study investigated how states curiosity and post-answer interest affect memory for answers to trivia questions in children and adolescents. Our results revealed that (1) both children and adolescents showed better memory for answers to questions associated with high curiosity. (2) Adolescents compared to children showed a greater memory enhancement when they found the answer more interesting. Consequently, answers associated with higher positive IPEs were better remembered by adolescents than by children. In particular, answers associated with higher positive IPEs were remembered by adolescents at levels comparable to their memory for information associated with high curiosity states, suggesting that higher than expected information value can offset the effects of lower states of curiosity in adolescence. (3) Finally, exploratory correlational analyses revealed that children and adolescents with higher curiosity-related memory enhancements for trivia answers showed higher curiosity-related spill-over effects on memory for incidental face images presented during the anticipation period.

### Curiosity Effects on Memory

Corroborating previous findings in adults (for a review, see Gruber et al., 2019), states of high curiosity were associated with better memory for trivia answers in children and adolescents. Curiosity is often considered a powerful tool in educational contexts (Engel, 2011). As many of our questions included educationally-relevant content, our results suggest that children and adolescents who are curious about a question are indeed more likely to remember the associated answer. These results complement previous findings that trait curiosity affects cognition (for reviews, see Grossnickle, 2016; Renninger & Hidi, 2019). Specifically, studies have consistently demonstrated that higher trait curiosity is positively associated with academic achievement in children (Kashdan & Yuen, 2007; Shah et al., 2018; von Stumm et al., 2011). At the same time, the present results elucidate a different aspect of curiosity, namely states of curiosity. While state and trait curiosity are thought to be closely related (Baranes et al., 2015; Grossnickle, 2016; Risko et al., 2012), characterizing the development of state curiosity entails clear benefits in that it can help tailor strategies and interventions to optimally stimulate states of curiosity across childhood and adolescence (Hassinger-Das & Hirsh-Pasek, 2018; Jirout et al., 2018; Kashdan & Yuen, 2007; Shah et al., 2018). Interestingly, these studies have also revealed that the relation between trait curiosity and academic achievement depends on additional factors such as children’s perception of the school situation (Kashdan & Yuen, 2007) or socio-economic status (Shah et al., 2018). Future studies are needed to examine if state effects of curiosity on learning in children interact similarly with external constraints.

Extensive research in infants and young children has demonstrated that there are tight links between the level of uncertainty of knowledge gaps and exploratory behavior (Kidd & Hayden, 2015; Schulz & Bonawitz, 2007). Here, we extend these findings to episodic memory and show that states of increased curiosity enhance memory for information associated with these states in children and adolescents. Of note, we found curiosity-related memory enhancements in an immediate memory test whereas curiosity-related memory benefits in adults have also been demonstrated after longer delays (Gruber et al., 2014; Kang et al., 2019; Stare et al., 2018). Future studies should investigate curiosity-related memory persistence in children and adolescents over extended periods of time.

### Interest and IPE Effects on Memory

Post-answer interest effects on memory for trivia answers were more pronounced in adolescents than in children. Post-answer interest effects have been shown to largely mediate the effects of pre-answer curiosity on memory in adults (Fastrich et al., 2018), and the present findings suggest that a similar pattern is present in adolescents, but not in children. These age differences are unlikely to reflect lower interest in children, as our sample did not show differences in average interest between children and adolescents. Furthermore, variation in positive IPEs modulated the effects of curiosity on children’s memory to a lesser degree than in adolescents. These results suggest that children’s learning may be strongly guided by their expectations rather than the value of information. Although future research is needed to test specific accounts of the development of situational interest on memory, our findings are consistent with several frameworks, and provide first indications about how these mechanisms may result in different effects of situational interest across childhood and adolescence.

#### (1) Interest and IPE effects on memory might develop similarly to reward processes

Based on the information-as-reward hypothesis, which posits that curiosity conforms to basic characteristics of reward-motivated behavior (Marvin & Shohamy, 2016), there are clear parallels between our results and findings from reinforcement learning. More specifically, children in the present study may be less sensitive to changes in the value of the outcome than adolescents. Theoretical models in reinforcement learning (Daw, Niv, & Dayan, 2005) suggest that individual choices and the adaptive control of behavior depends on the interplay between two systems. Model-free learning is rapid and habitual, and depends on choice value which is updated via trial and error. In contrast, model-based learning is more demanding and entails constructing and searching through a cognitive model of potential state transitions and outcomes in order to select an action or to make a choice. Model-based learning is thus characterized by increased sensitivity to changes in contingencies between actions and outcomes, and to changes in the value of the outcome. Interestingly, recent evidence suggests that children are less likely than adolescents to engage in model-based learning and rely more strongly on model-free learning (Decker et al., 2016). These and other results (for a review, see Nussenbaum & Hartley, 2019) suggest that children may be less sensitive to changes in outcome value in the context of reward-based learning. If the effects of the discrepancy between states of curiosity and interest are similar to discrepancy in expectation and outcome in reward-based learning, this might explain why children show less memory benefits from post-answer value updating (measured via post-answer interest and IPEs) compared to adolescents.

On the other hand, adolescence is a period marked by changes in motivated behavior and increased sensitivity to rewards (Galvan et al., 2006; van Duijvenvoorde et al., 2016). While increased reward sensitivity has been repeatedly associated with increased risk-taking behavior, it has also been shown to positively affect cognitive functioning (Davidow et al., 2016; Geier & Luna, 2012; van den Bos et al., 2012). To date, the effects of intrinsic rewards in adolescence have rarely been examined (e.g., Satterthwaite et al., 2012). The present study corroborates and extends previous research by demonstrating that larger interest and positive IPEs associated with particular information can positively bias episodic memory, and that these memory-enhancing effects increase in the transition into adolescence, potentially due to increased reward sensitivity. The interpretation is consistent with findings of increased magnitude of prediction error signals during reinforcement learning in adolescence (Cohen et al., 2010; Hauser et al., 2015). Our findings on IPEs on memory in adolescents are in line with accumulating findings on the positive effects of reward prediction errors (i.e., received reward - expected reward) on episodic memory in adults (for a review, see Ergo et al., 2020). They are also in line with recent studies in adults showing that prediction errors about information enhance episodic memory (Greve et al., 2017) and that prediction errors about semantic facts have a positive effect on episodic memory via recruitment of the striatum (Pine et al., 2018). However, the neural mechanisms of IPEs in a trivia paradigm have not been directly investigated. It is therefore not known how the neural mechanisms associated with IPEs and other prediction errors, especially reward prediction errors, precisely map onto each other.

#### (2) Surprise as a potential mechanism underlying the effects of interest and IPE on memory

Alternatively, our IPE findings might be explained by surprise about the content of the information. It has been shown that actively generating predictions about answers to trivia enhances curiosity and learning (Brod et al., 2018; Brod & Breitwieser, 2019) such that the generation of predictions about possible answers leads to larger surprise signals as indexed by pupil dilations (Brod et al., 2018). These results point to an alternative interpretation for our IPE findings to the interpretation drawn from the reward prediction error findings above. That is, the stronger positive effects of IPEs on memory in adolescents may be driven by a stronger surprise in adolescents. Although we did not directly assess how IPEs are related to surprise, we can only speculate whether the observed positive effects of IPEs on memory in adolescents are perhaps driven by a stronger surprise in adolescents. Compared to children, adolescents have more efficient executive control (Amso & Scerif, 2015; Crone, 2009) and might engage in stronger predictions about the potential trivia answers. Consistent with this speculation, a study in children has shown that wrong predictions about potential events enhanced learning only in children with higher executive control (Brod et al., 2019). Taken together, the positive effects of interest and IPEs on memory might depend on the degree of available executive control to facilitate active predictions about the expected outcome, and thereby lead to stronger surprise and better memory. It should be noted, however, that one recent study in adults used computationally modelling to show that the enhancing effects of reward prediction errors on memory were not influenced by surprise (Jang et al., 2019). As reward prediction errors depend more on dopaminergic activity whereas surprise depends more on noradrenergic activity (Schultz, 2013; Yu & Dayan, 2005), future neuroimaging studies to assess the neural mechanisms of post-answer processes may help dissociate whether the effects of IPEs on memory are related to dopaminergic prediction errors or surprise-dependent noradrenergic responses in children and adolescents. Furthermore, future studies utilizing finer-grained or continuous scales should be used to achieve a better understanding of how the precise degree of positive or negative discrepancy between curiosity and interest results in differential memory benefits.

#### (3) Cognitive vs. affective processes might contribute to the effects of interest and IPEs on memory in children and adolescents

A more nuanced interpretation of the observed interest-and IPE-related memory enhancements for adolescents could be that surprise was elicited quantitatively to the same extent across children and adolescents (which our findings support by not showing a difference in average interest and IPE ratings between children and adolescents), but was characterized by qualitatively different components. The influential 4-phase model of interest development (Hidi & Renninger, 2006) proposes that situational interest triggers early affective processes, but during later stages of interest development affective processes subside and more cognitive processes dominate. In line with this model, a study by Frenzel and colleagues demonstrated qualitative changes in individual interest from childhood into adolescence (Frenzel et al., 2012) such that interest predominantly triggered affective components in children but mostly cognitive components in adolescents. Consistent with our above speculation that adolescents might engage in more proactive cognitive components (i.e., specific predictions about trivia answers), the larger interest- and IPE-related memory enhancements in adolescents might be explained by more cognitive than affective components that are initiated by the presentation of the trivia answers. These cognitive components in turn may facilitate learning to a greater degree, resulting in better memory in adolescents. In contrast, potentially a greater engagement of post-answer affective processes in children might only have a little effect on memory. In addition, children may not have yet developed the cognitive capacities to efficiently update their attentional resources to information that is more interesting than originally expected. Such an interpretation is consistent with the protracted development of prefrontal executive functioning in childhood (Amso & Scerif, 2015; Crone, 2009; Zelazo & Carlson, 2012) and their role for model-based learning (Decker et al., 2016).

While the different theoretical accounts reviewed above propose different mechanisms by which interest and IPE may affect learning in childhood and adolescence, they share the common notion that developmental differences may be related to the protracted development of executive control and the underlying prefrontal brain regions (Amso & Scerif, 2015; Crone, 2009). While this idea has to be tested directly in future research, it aligns well with the recently proposed Prediction, Appraisal, Curiosity, and Exploration (PACE) framework (Gruber & Ranganath, 2019). The PACE framework might help synthesize the above explanations for the IPE effects on memory as it differentiates the respective contributions of the prefrontal cortex, the striatum, and the hippocampus in support of how prediction errors lead to curiosity-related memory enhancements (Gruber & Ranganath, 2019). Perhaps most relevant for development, PACE proposes that an appraisal process supported by prefrontal cortex evaluates whether hippocampus-related prediction errors lead to striatal-related activity stimulating increases in information-seeking and in memory. Thus, the PACE framework (Gruber & Ranganath, 2019) would predict that children might show a lack of or more variable appraisal due to protracted prefrontal development in the service of learning and memory (Fandakova et al., 2017, 2018). Information that elicits higher prediction errors (such as more interesting than initially expected information in our study) might contribute to weaker effects on how interest and IPEs influence memory in children with less reliable appraisal processes. The idea of less prefrontal appraisal in children would generally be consistent with ideas that interest relies more on affective than cognitive components in children (cf. Frenzel et al., 2012). It would also be consistent with the information-as-reward hypothesis as it has been shown that prefrontal input affects dopaminergic responses for motivated behavior (Ballard et al., 2011; Frankle et al., 2006; Lisman & Grace, 2005) and it is also well aligned with the possible involvement of surprise as memory for surprising, unexpected information has been shown to depend on the successful communication of prefrontal cortex with the hippocampus and striatum (Gruber et al., 2018; Lisman & Grace, 2005; Murty & Adcock, 2014).

### Curiosity-related memory enhancements for incidental face images

In adults, curiosity states also enhance memory for incidental face images encountered during high- compared to low-curiosity states (Galli et al., 2018; Gruber et al., 2014; Stare et al., 2018). In contrast to our prediction, we did not observe that high-curiosity states increased memory for incidental face images in children and adolescents. One potential explanation for this null finding is that although we found significant curiosity-related memory enhancements for trivia answers, the magnitude (∼7%) was smaller than in comparable studies with young adults (∼18%) (Gruber et al., 2014; Stare et al., 2018), potentially reflecting a wider variation in curiosity stimulation across children and adolescents and thereby resulting in a lower average memory benefit than that observed in young adults. This interpretation is consistent with the exploratory correlational analyses, which suggested that children demonstrating a greater curiosity-related memory benefit for trivia answers also tended to show a curiosity-related benefit for incidental information. Furthermore, memory performance for incidental faces in our sample was higher than in previous adult studies (Gruber et al., 2014; Stare et al., 2018). As spillover effects on memory have been shown to be only evident for weakly encoded items (Dunsmoor et al., 2015), encoding of the face images in the present study might have been too strong to isolate spillover effects. Finally, Gruber and colleagues (2014) showed that curiosity-related spillover effects depend on individual differences in activity and functional connectivity between reward- and memory-related regions. Future studies can address how variability in these regions is related to curiosity spillover effects on incidental memory in children and adolescents.

In conclusion, curiosity enhances memory in children and adolescents. Moreover, adolescents - but not children - showed an additional memory benefit when they found the information more interesting than expected, thereby counteracting the effects of low curiosity on later memory. As teachers need to spark students’ motivation to learn, these results indicate that states of pre-information curiosity and post-information interest play a critical role in learning and can be effectively harnessed as a tool in educational settings. Importantly, different strategies to trigger curiosity and interest in the classroom may result in distinct memory benefits across child development.

## Figures and Tables

**Table 1:** Model results from a mixed-model logistic regression in which trial-based accuracy of trivia answer recall was predicted by curiosity ratings, IPE and participants’ age.

	Estimate	Est. Error	lower-95% CrI	upper-95% CrI	Post. Prob
IPE	0.17	0.04	0.09	0.25	1*
Curiosity	0.35	0.05	0.25	0.44	1*
Age	0.11	0.07	-0.03	0.25	0.94
IPE x Curiosity	-0.05	0.03	-0.12	0.01	0.94
IPE x Age	0.04	0.03	-0.01	0.09	0.93
Curiosity x Age	0.04	0.03	-0.02	0.10	0.9
IPE x Curiosity x Age	-0.04	0.02	-0.08	0.00	0.97*
Low-curiosity Questions					
IPE	0.18	0.05	0.07	0.28	1*
Age	0.08	0.07	-0.07	0.22	0.85
IPE x Age	0.07	0.03	0.00	0.14	0.98*
High-curiosity Questions					
IPE	0.03	0.06	-0.09	0.16	0.7
Age	0.16	0.08	0.01	0.31	0.98*
IPE x Age	0.01	0.04	-0.08	0.09	0.56

Note: Estimates are on the log-odds scale.

* Two-sided hypothesis, the value tested against lies outside the 95%-CI. Est. Error = estimated error; CrI = credible interval; Post. Prob = posterior probability

**Table 2:** Model results from a mixed-model logistic regression in which trial-based accuracy of trivia answer recall was predicted by curiosity ratings, post-answer interest ratings and participants’ age.

	Estimate	Est. Error	lower-95% CrI	upper-95% CrI	Post. Prob
Interest	0.17	0.04	0.09	0.25	1*
Curiosity	0.17	0.04	0.09	0.25	1*
Age	0.15	0.07	0.01	0.29	0.98*
Interest x Curiosity	-0.09	0.04	-0.17	-0.02	0.99*
Interest x Age	0.04	0.03	-0.01	0.09	0.92
Curiosity x Age	0.00	0.03	-0.05	0.05	0.5
Interest x Curiosity x Age	-0.02	0.02	-0.07	0.02	0.81

Note: Estimates are on the log-odds scale.

* Two-sided hypothesis, the value tested against lies outside the 95%-CI. Est. Error = estimated error; CrI = credible interval; Post. Prob = posterior probability

**Table 3:** Mean (standard deviations) memory accuracy (hits – false alarms) for incidental face images presented during high-curiosity (i.e., “3” & “4” ratings) and low-curiosity (i.e., “1” & “2” ratings) states.

	High curiosity	Low curiosity
Children	49.6% (22.3%)	49.7% (23.7%)
Adolescents	58.3% (13.1%)	57.3% (16.0%)

## Research Highlights

▪ We examine the effects of pre-information curiosity and post-information interest on memory in children and adolescents
▪ States of high curiosity enhance learning and memory in children and adolescents
▪ Adolescents, but not children, were more likely to remember information when it was more interesting than their initial state of curiosity
